# Ruptured Liver Abscess in Neonates: Report of Two Cases

**DOI:** 10.21699/jns.v5i3.335

**Published:** 2016-07-03

**Authors:** Niyaz Ahmed Khan, SR Choudhury, Praveen Jhanwar

**Affiliations:** Department of Paediatric Surgery, Kalawati Saran Children's Hospital, Lady Hardinge Medical College, New Delhi

**Keywords:** Neonatal hepatic abscess, Parietal wall, Rupture

## Abstract

Neonatal hepatic abscess is a rare disease seen mainly in preterm following umbilical catheterisation. Liver abscess in term neonates without any predisposing factor is still rarer and only few cases have been reported in the literature. Here we report two cases of liver abscess in term neonates presenting with abdominal mass due to rupture.

## INTRODUCTION

Neonatal liver abscess is rare entity and till date fewer than 100 cases have been reported in literature. Generally they occur in preterm infants with certain risk factors like umbilical vein catheterization [1]. Treatment consists of aspiration, drainage of abscess with antibiotics [2]. Presentation in a full tern new born with an abdominal mass due to ruptured liver abscess into parietal wall has not been reported earlier.

## CASE SERIES

**Case 1:**

 A 26-day old female full term neonate was admitted to the surgical ward with history of swelling in the left upper abdomen (Fig.1) and fever of 5 days duration. Apart from leukocytosis (TLC 29,700/cu.mm), there was no sign of overt sepsis with CRP being negative. Ultrasound abdomen revealed 30×50×20 mm hypoechoic lesion in the left lobe of liver, with capsular breach and exophytic extension to the anterior abdominal wall (Fig.2). Liver functions tests and coagulation profile were normal. Intra-venous antibiotics with ceftriaxone and metronidazole were started and a cruciate incision was given for drainage of abscess from the parietal wall. Culture of pus from the abscess did not grow any organism. Post drainage recovery was uneventful and the patient was discharged after receiving 5 days of intra venous antibiotics which was switched to oral antibiotics for a further period of total three weeks. Follow-up at 6 weeks did not show any evidence of recurrence with ultrasound revealing a 5X2x2 mm residual cavity. 

**Figure:**
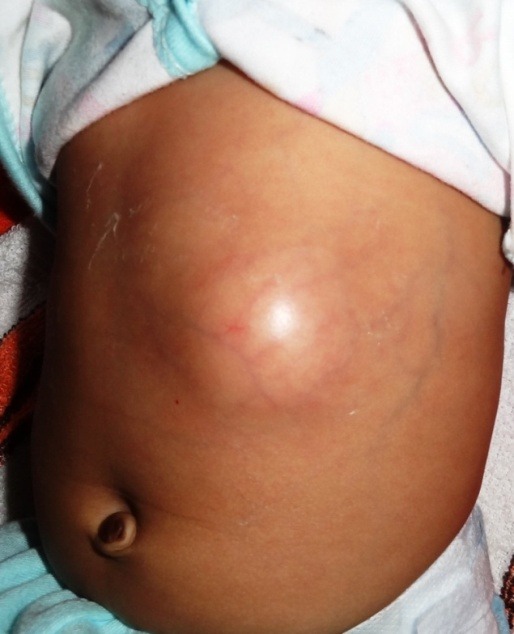
Figure 1: Case 1- presenting with inflammatory swelling in the left upper abdomen.

**Figure:**
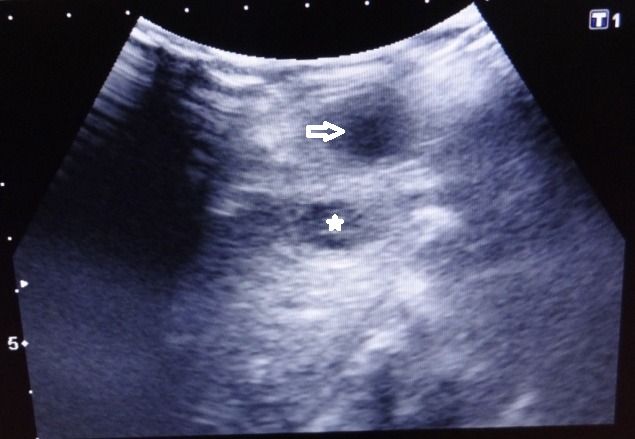
Figure 2: Case 1- USG showing hepatic abscess (asterisk) with extension to abdominal wall (arrow).

**Case 2:**

A 1-month old male child born by full term normal vaginal delivery with no history of umbilical catheterization, presented to surgical clinic with a right upper abdominal swelling of one week duration. Total leukocyte counts were 12,500/ cu.mm and CRP being negative. Ultrasound abdomen revealed a heterogeneous mass lesion in right lobe of liver segment V of size 55×34 mm with a 31×14 mm exophytic extension in the parietal wall. Computerized tomography (CT) scan showed a hypo dense multiloculated lesion of 55×34 mm in size in the right lobe of the liver with exophytic cystic component projecting into the abdominal wall measuring 32×15mm and showing thick peripheral rim enhancement (Fig. 3). Liver function tests were normal. Alpha- fetoprotein level was 607.70 IU/ml. Aspiration of the parietal wall swelling revealed thick pus of about 15cc volume. Intravenous antibiotics with combination of ceftriaxone and metronidazole were started and subsequent pus culture showed growth of methicillin resistant Staphylococcus aureus sensitive to vancomycin and linezolid. A cruciate incision was given over the parietal swelling and drainage of the abscess was done and intravenous vancomycin was added. Patient recovered after drainage and was discharged on oral linezolid for further two weeks. A review ultrasound at 3 months showed near complete resolution of the lesion.

**Figure:**
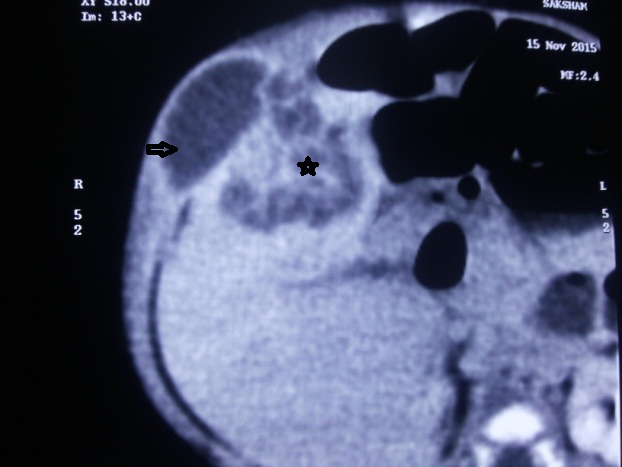
Figure 3: Case 2- CT scan showing hepatic abscess (asterisk) with extension to abdominal wall (arrow).

## DISCUSSION

Neonatal hepatic abscess is generally seen in premature neonates who undergo umbilical vein cannulation. Systemic sepsis, necrotizing enterocolitis, and central parenteral nutrition catheters are the other risk factors.[3,4] The causative agents for neonatal liver abscess have been variable. Gram negative enteric bacteria and Staphylococcus aureus are the most common organisms isolated [5]. Rarer organisms like Candida [6], Serratia are generally seen in premature neonatal hepatic abscess. In one of our cases the culture grew methicillin resistant Staphylococcus aureus (MRSA) indicating extraneous source of infection. Community acquired MRSA (CA-MRSA) is one of the emerging pathogen which unlike nosocomially acquired MRSA (HA-MRSA), affects the children who are not exposed to health care system [7].

The clinical features of these neonates are very different from infants and children. Non-specific signs like delayed capillary refill time, refusal to feed, abdominal distension are reported in neonates; thus making the diagnosis difficult. In our cases, the presentation was with abdominal wall swelling. Ultrasound abdomen is one of the main tools for the diagnosis. However sometimes it is difficult to distinguish liver abscess from other hepatic masses like hepatoblastoma, infantile hemangioendothelioma or hamartoma where computerized tomography scan may be helpful [8].

Complications like rupture of liver abscess into peritoneum, pleural cavity or portal venous thrombosis are rare in neonates. Till date no cases of rupture of neonatal liver abscess into parietal wall have been reported in the literature.

Treatment of hepatic abscess has evolved from open surgical drainage [9] to more conservative imaging guided percutaneous aspiration, and antibiotics therapy [2]. However, the ideal management still remains controversial. One study on neonatal hepatic abscess showed imaging guided aspiration or drainage has a good long term outcome [2]. Medical management with intravenous antibiotics is crucial and should be started at the earliest and must be guided according to the sensitivity pattern. Duration of antibiotics should be for a minimum period of three weeks [10].

To conclude, rupture of liver abscess in to the anterior abdominal wall in full term neonates without any predisposing factor is a rarity. Diagnosis can be aided by clinical examination, imaging and aspiration. Outcome is favourable when managed by adequate drainage with appropriate antibiotics.

## Footnotes

**Source of Support:** Nil

**Conflict of Interest:** None
